# Multi-response optimization of *Artemia* hatching process using split-split-plot design based response surface methodology

**DOI:** 10.1038/srep40394

**Published:** 2017-01-16

**Authors:** V. V. Arun, Neelam Saharan, V. Ramasubramanian, A. M. Babitha Rani, K. R. Salin, Ravindra Sontakke, Harsha Haridas, Deepak George Pazhayamadom

**Affiliations:** 1 ICAR-Central Institute of Fisheries Education, Mumbai, India; 2 Aquaculture and Aquatic Resources Management, Asian Institute of Technology, Thailand; 3 Department of Primary Industry and Fisheries, Northern Territory Government, Australia

## Abstract

A novel method, BBD-SSPD is proposed by the combination of Box-Behnken Design (BBD) and Split-Split Plot Design (SSPD) which would ensure minimum number of experimental runs, leading to economical utilization in multi- factorial experiments. The brine shrimp *Artemia* was tested to study the combined effects of photoperiod, temperature and salinity, each with three levels, on the hatching percentage and hatching time of their cysts. The BBD was employed to select 13 treatment combinations out of the 27 possible combinations that were grouped in an SSPD arrangement. Multiple responses were optimized simultaneously using Derringer’s desirability function. Photoperiod and temperature as well as temperature-salinity interaction were found to significantly affect the hatching percentage of *Artemia*, while the hatching time was significantly influenced by photoperiod and temperature, and their interaction. The optimum conditions were 23 h photoperiod, 29 °C temperature and 28 ppt salinity resulting in 96.8% hatching in 18.94 h. In order to verify the results obtained from BBD-SSPD experiment, the experiment was repeated preserving the same set up. Results of verification experiment were found to be similar to experiment originally conducted. It is expected that this method would be suitable to optimize the hatching process of animal eggs.

Aquaculture experiments often adopt a unifactorial approach i.e. the effect of a single factor (e.g. photoperiod, temperature or salinity) is studied at a time keeping other factors constant^1^. However, aquatic organisms live in a complex environment where multiple factors interact with one another, affecting their survival and growth. Therefore, multifactorial experiments would reveal not only the effect of individual factors but their interaction effects as well^2^,^3^.

The branchiopod crustacean, *Artemia*, inhabiting hypersaline water produces dormant cysts under unfavorable environment. These cysts would hatch into nauplii when conditions become favorable. The newly hatched nauplii of *Artemia* form an excellent live feed in finfish and shellfish hatcheries^4^. While the hatching process of dormant *Artemia* cyst is critically affected by several abiotic factors^5^,^6^,^7^, the most significant among them are photoperiod, temperature and salinity^7^. The peculiar cyst formation and hatching properties qualify *Artemia* as an excellent model organism to study the hatching process of animal eggs.

Multifactorial experiments become difficult to organize, particularly when certain factors cannot be randomized easily across the experimental units because of practical inconvenience and economic constraints. In such situations, the split-plot design is a good choice where a hard-to-change (HTC) factor is applied to a group of experimental units (or blocks)^8^,^9^. When compared to Completely Randomized Design (CRD), the split-plot design would give more precise results at lower cost^8^,^10^.

Simultaneous optimization of the factors is also necessary to achieve the desired outputs in multifactorial trials. Response surface methodology (RSM), which is a collection of statistical and mathematical techniques is useful to develop, improve, and optimize the processes^11^. RSM helps in development of an adequate functional relationship between a response of interest and input variables^12^. It has got varied applications in the industry (like food processing, engineering, etc.) as well as in research. In RSM, three level factorial designs such as the Box-Behnken Design (BBD) could be used to investigate both individual and interactive effects of input variables with minimum experimental runs^11^. Further, as desirable output of a process is always a function of several responses, hatching percentage and hatching time have to be optimized simultaneously. In such cases simultaneous optimization technique called desirability function can be used for optimizing multiple responses, wherein a “score” is assigned to the set of responses and factor settings such that they are chosen to maximize that score^11^. However the inputs supplied to this function will be the responses obtained at various levels of independent variables by conducting BBD-SSPD experiment described subsequently. Thus, RSM can be used to find the optimum (and robust) operating conditions so that the maximum hatching output can be ensured when various abiotic factors have their effect on the hatching performance^13^,^14^,^15^,^16^. However, there is lack of information on the designs for the simultaneous optimization of multiple responses which can be employed to investigate biological processes with minimum experimental runs and randomization restriction in a cost-effective manner.

We propose a novel method BBD-SSPD by combining Box-Behnken Design (BBD) and Split-Split Plot Design (SSPD) to optimize the hatching process of *Artemia* cysts. The novelty of this method lies on two aspects. Firstly, we believe that this method is used for the first time in a biological experiment where the conditions are quite dynamic compared to the similar applications in other fields such as engineering wherein the conditions are relatively easier to control. Secondly, even under such a highly restricted randomization experiment, the use of desirability function has been demonstrated for simultaneous optimization of the two responses using BBD-SSPD. This method specifically can be applied in the breeding and hatching of commercially important finfish and shellfish used in aquaculture. The proposed method can potentially be used to optimize the hatching process of egg layers like birds, reptiles, and aquatic animals that are of high economic value. The present study is the first attempt for the simultaneous optimization of factors such as photoperiod, temperature and salinity on the hatching process of *Artemia* cysts, using BBD-SSPD to achieve the maximum hatching percentage within the minimum hatching time.

## Results

### Hatching Percentage

Photoperiod and temperature were found to be the most important factors affecting the hatching percentage of *Artemia*. The application of RSM resulted in the following regression equation which explains the functional relationship between hatching percentage and process variables in coded units (Equation 1). The regression model was reduced using the process of backward elimination where the non-significant terms were removed. While the linear and quadratic effects of salinity had no significant (P > 0.05) effect on hatching percentage, these were retained to preserve the hierarchy of the fitted model.


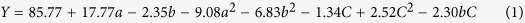


where Y, a, b and C represent hatching percentage, photoperiod, temperature and salinity respectively. Factors in capital letters indicate ETC (Easy To Change) factor. The goodness of fit of the model was verified using determination coefficient (R^2^) where the present model explained 99% of the variation in the observations (R^2^ = 0.99). Adjusted determination coefficient (Adj R^2^ = 0.99) had also reiterated the significance of the model. Results of Restricted Maximum Likelihood (REML) analysis for the selected model are given in Table 1.

**Figure 1:**
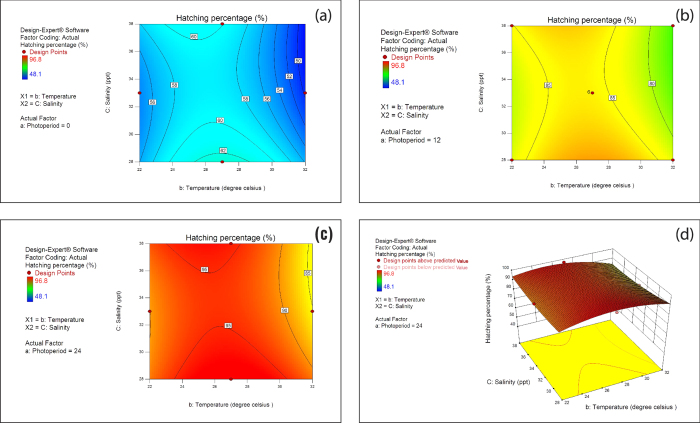
Contour plot and Response surface plot of the hatching percentage (**a**) Contour plot of the hatching percentage obtained at 0 h photoperiod (**b**) Contour plot of the hatching percentage observed at 12 h photoperiod (**c**) Contour plot of the hatching percentage observed at 24 h photoperiod. Numbers shown in the square brackets are the hatching percentage at the corresponding contour (**d**) Response surface plot of hatching percentage (%): Effect of temperature and salinity at 24 h photoperiod. The bottom yellow plane represents the contour plot of the hatching percentage observed at 24 h photoperiod. Red dots in the axis show the levels of treatments used in the present study. Factors in capital letters indicate ETC factors.

The linear effect as well as the quadratic effect of photoperiod was significant (*P* < 0.0001, Table 1). The hatching percentage was significantly affected by the linear effect of temperature (*P* < 0.01, Table 1) as well as the quadratic effect of temperature (*P* < 0.0001, Table 1). The interaction between temperature and salinity was also found to play a significant (*P* < 0.05, Table 1) role in the hatching percentage of *Artemia*. Contour plots show the temperature and salinity interaction (Fig. 1a,b,c) under each photoperiod as well as their associated effect on hatching percentage. The center point or stationary point of the contour plot represented in Fig. 1a,b,c is a saddle point. In the contour plots, as the photoperiod increases from 0 to 24 h, hatching percentage also increases from 48.1 to 96.8%, which indicates that hatching percentage of *Artemia* was strongly affected by darkness (0 h), and that the effect of light was negligible in the later stages of incubation of cysts. The low-high interaction between photoperiod and temperature caused drastic reduction in hatching percentages.

Figure 1d represents 3D response surface plot of hatching percentage depicting interaction of temperature and salinity at 24 h photoperiod. It is evident from the plot that the hatching percentage was highly influenced by photoperiod as described in Table 1. The highest hatching percentage (>90%) could be achieved at 24 h photoperiod, 28 ppt salinity and temperature above 27 °C. Flat surface (less curvature of salinity axis compared to temperature axis) plot showed the minimal effect of salinity (Fig. 1d). However, it is interesting to note that even though the effect of salinity on hatching percentage was not statistically significant, still it strongly influenced the hatching percentage as observed from the surface plot (Fig. 1d).

### Hatching time

The effect of independent variables on hatching time could be represented using a regression equation in coded units (Equation 2). The regression model was reduced using the process of backward elimination where the non-significant terms were removed. The salinity term was retained in the model to maintain the hierarchy.


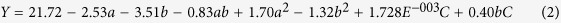


where Y, a, b and C represent hatching time, photoperiod, temperature and salinity, respectively. Factors in capital letters indicate ETC factors. The value of the determination coefficient (R^2^ = 0.98) indicates that only 2% of the variation in the response is not explained by the model. The high value of the adjusted determination coefficient (Adj R^2^ = 0.95) also suggests that the model could be used for predictions. REML analysis results are given in Table 1.

**Figure 2:**
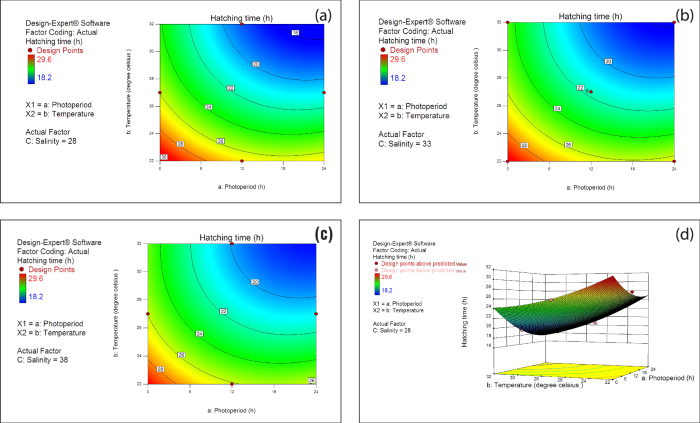
Contour plot and Response surface plot of the hatching time (**a**) Contour plot of the hatching time observed at 28 ppt salinity (**b**) Contour plot of the hatching time observed at 33 ppt salinity (**c**) Contour plot of the hatching time observed at 38 ppt salinity. Numbers shown in the square brackets represents the hatching time at the corresponding contour. (**d**) Response surface plot of hatching time (h): Effect of temperature and photoperiod at 28 ppt salinity. The bottom yellow plane represents the contour plot of the hatching percentage observed at 28 ppt salinity. Red dots in the axis show the treatments level used in the present study. Factors in capital letters indicate ETC factor.

The central point in the contour plots of photoperiod and temperature interaction (Fig. 2) represented in the rectangles of each contour plot is characterized by a rising ridge system. It could be observed from the contour plot that hatching time was more sensitive to unit changes in photoperiod compared to temperature especially at lower temperatures (Fig. 2). The blue coloured inner contours of the rising ridge represents the desirable minimum hatching time (<20 h). The variation in colour from the outer red to inner blue at 28, 33 and 38 ppt salinity has nearly similar contour distribution, which reveals negligible effect of salinity on hatching time compared to the photoperiod-temperature interaction. Lower levels of photoperiod (0 h) showed strong negative effect on the hatching time compared to the medium (12 h) and high level (24 h) (Fig. 2b).

Surface plot of fitted quadratic equation of hatching time is presented in the Fig. 2d, which indicates that the minimum hatching time for *Artemia* was achieved under constant illumination (24 h photoperiod). At this photoperiod, the hatching time was apparently lowered by the higher temperature (above 30 °C) and salinity (above 33 ppt) levels.

Hatching time was highly influenced by the linear effect of photoperiod and temperature (*P* < 0.0001, Table 1), while quadratic effect of the photoperiod and temperature as well as their interaction had also affected the hatching time significantly (*P* < 0.05, Table 1). All of these provide conclusive evidence that the hatching time of *Artemia* is more influenced by photoperiod and water temperature than by the salinity of cyst incubation.

In order to verify the result obtained in the present study, a verification experiment has been conducted in which *Artemia* cysts were hatched under similar conditions of the original experiment. Hatching percentage was significantly affected by linear and quadratic effects of both photoperiod and temperature (*P* < 0.05) as well as temperature - salinity interaction (*P* < 0.05). Hatching time was significantly affected by photoperiod and temperature (*P* < 0.05) and their interaction (*P* < 0.05). In single factorial experiments for hatching, hatching percentage and hatching time were significantly affected by photoperiod (P < 0.05) and temperature (P < 0.05). Salinity was not having any significant effect on hatching of *Artemia* cysts.

### Desirability Function

**Figure 3:**
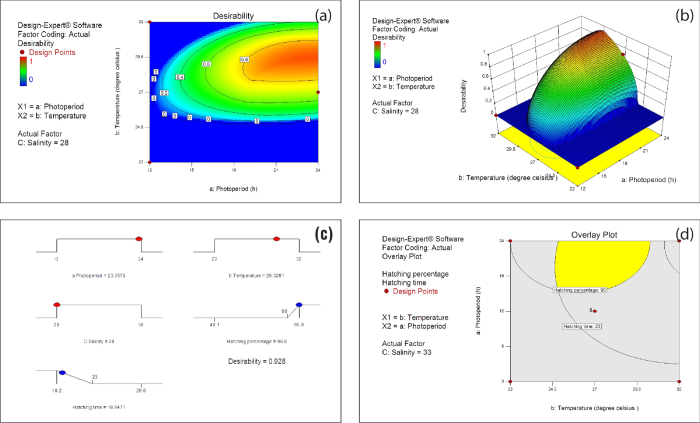
Desirability function and overlay plot (**a**) contour plot of desirability function: Effect of photoperiod and temperature at 28 ppt salinity. (**b**) Response surface plot of desirability function: Effect of photoperiod and temperature at 28 ppt salinity he bottom yellow plane represents the contour plot of the hatching percentage observed at 28 ppt salinity Numbers shown in the square brackets represents the desirability function at the corresponding contour. Factors in capital letters indicate ETC factors. (**c**) Desirability ramp (graphical representation of numerical optimization results) for achieving optimum hatching output (maximum hatching percentage and minimum hatching time). The optimum value of photoperiod, temperature and salinity to achieve an overall desirability of 0.928 is depicted in desirability ramp. (**d**) Overlay plot showing recommended hatching operation region (yellow colour) for achieving desired hatching percentage and hatching time (here minimum 90% hatching percentage and maximum 23 h hatching time is set to ensure economical operation.

The desirability function was calculated using the criteria given in Table 2 and the contour plot of desirability function is given in Fig. 3a. Maximum desirability that can be achieved is 1. Desirability above 0.8 is represented by red colored region of the contour plot. The desirability had increased with duration of photoperiod, registering the maximum of above 20 h as evident from Fig. 3a. However, the maximum desirability was achieved within a temperature range of 28–30 °C at 28 ppt salinity (Fig. 3a). Desirability was zero below 12 h photoperiod and below 25 °C temperature (Fig. 3a).

The maximum desirability is depicted by the top red portion of the dome shaped response surface plot of desirability (Fig. 3b). The flat dark blue portion is the zero desirability area. This surface plot combines the individual surface plots of both responses (hatching percentage and hatching time) based on the desirability criteria set for each. The curvature area satisfies the optimality criteria of both responses simultaneously. It can also be observed from the plot that the maximum desirability that could be achieved was around 0.9. An exact depiction of the operating condition and corresponding desirability could be obtained from the desirability ramp, a graphical representation of the numerical optimization results (Fig. 3c). This ramp could be utilized to find the optimum operating conditions (absolute value of photoperiod, temperature and salinity) and overall desirability to achieve optimum hatching output. Accordingly, the optimum conditions for achieving a maximum hatching percentage of 96.8 and minimum hatching time of 18.94 h were 23.35 h photoperiod, 29.3 °C temperature and 28 ppt salinity. Overall desirability achieved under optimum conditions was 0.928.

A final illustration of the optimized operating conditions of the hatching process of *Artemia* was obtained using an overlay plot (Fig. 3d) created by superimposing the contour plots of individual responses. The overlay plot could be visually searched for the best compromise of operating conditions that ensured the maximum hatching percentage and minimum hatching time. The yellow region in Fig. 3d shows the optimum region for hatchery operations highlighting the minimum hatching percentage of 90% and maximum hatching time of 23 h is ensured.

## Discussion

Most of the earlier reports testing the effect of abiotic factors on the hatching process of *Artemia* were unifactorial experiments^1^. However, it is evident from the present study that the factors (such as temperature, photoperiod or salinity) would interact with one another and in turn affect the hatching percentage differently. In this study, we found that photoperiod and temperature were the most significant factors (Table 1) in determining the optimum hatching percentage and hatching time compared to salinity of the ambient water.

In the present study, photoperiod, temperature and salinity were selected due to their practical significance in hatchery operations^17^. In a hatchery, temperature may vary according to seasonal changes in the weather. Most of the other parameters such as pH, dissolved oxygen level etc. can be controlled easily in a hatchery. The second degree quadratic equation depicting the functional relationship between hatching percentage and abiotic factors could be used to predict the hatching percentage of *Artemia* with changing environmental parameters. Thus by keeping other abiotic factors constant, the model developed in the present study could be used for predicting the hatching percentage of *Artemia* cysts.

In a previous study, the hatching percentage of *Artemia franciscana* reported at 28 °C and 24 h photoperiod was 88.24 ± 3.6%^18^. Similarly, the hatching percentage reported for *Artemia* cysts collected from Namibia was 90.76 ± 1.85% at 25 °C and 24 h photoperiod^19^. The experimental conditions in these two studies were similar to the optimized conditions derived in the present study (23 h photoperiod; 29 °C temperature; 28 ppt salinity; and 90% hatching, as evident from Fig. 3d), except in the case of salinity, the effect of which was found to be non-significant in the latter.

We found that photoperiod plays a key role in the development rate of *Artemia* embryos. Thus, hatching percentage was drastically affected by even small changes in the photoperiod and temperature showing highly significant linear and quadratic effects (Table 1). The hatching percentage of California strain of *Artemia*, which was reported to be 62%^20^ under darkness had drastically improved to 95% when it was exposed to 24 h photoperiod at a temperature of 28 °C. Similarly in the present study, the hatching percentages obtained under darkness and 24 h photoperiod were 63.2% and 96.8%, respectively at 27 °C (Table 3). It is also evident from Fig. 1a,b,c that the hatching percentage improved with increase in photoperiod from 0 h to 24 h, and this trend is confirmed by the previous reports. However, it could be observed from the contour plot that during the later stages of incubation, the effect of light was minimal as compared to the 0 h photoperiod (Fig. 1a,b,c).

Even a minimal change in temperature had also resulted in substantial variation in the hatching percentage. This could be attributed to the highly significant linear as well as quadratic effect of temperature on hatching percentage (Table 1). Hatching of brine shrimp eggs is a temperature dependent process which gets accelerated with rising temperature within the biological optimal range of temperature^21^. *Artemia* cysts used in the present study had originated from the Great Salt Lake of USA. The hatching percentage of Great Salt Lake cysts of *Artemia*, among other strains was reported to decrease with rise in temperature from 25 °C to 30 °C^6^. For example, in Macau strain of *Artemia*, hatching percentage was reduced from 84.5% to 77.9% as the temperature was increased from 25 °C to 30 °C. Similar trend can be observed from Fig. 1c in the present results, in which the hatching percentage was reduced from 95% to 90% with a rise in temperature from 25 °C to 30 °C. It is evident from the present study that the hatching percentage was modulated in tune with the interaction between temperature and salinity, which might be attributed to the greater evaporation rates and consequent salinity at higher temperature. This effect might have been more pronounced because of the smaller volume of the experimental bottles used in this study. However, this holds true in such field conditions where hatching takes place in shallow water saltpans. This inference is also supported by a previous report that demonstrated significant interaction between temperature and salinity on survival and growth of various strains of *Artemia*^3^. However, salinity levels within the range of 28–38 ppt did not seem to have any significant effect on the hatching percentage (Table 1), which supports the general understanding that compared to the temperature and photoperiod, a broader range of salinity (15–35 ppt) can be used to achieve optimal hatching of *Artemia*^17^.

The mathematical model of hatching time derived in the present study could explain 98% of the variations observed in the experimental data. The prediction capacity of this model was also satisfactory as evidenced by the adjusted determination coefficient (Adj R^2^ = 0.95).

In a previous study, the hatching time reported was 20.5 h and 21.7 h for cysts collected from San Francisco Bay and Great Salt Lake, respectively where the cysts were hatched at 24 h photoperiod and 25 °C in sea water^22^. In the present study, hatching time was nearly 23 h when the temperature was around 25 °C (Fig. 2a,b,c). Similar to the case of hatching percentage as reported previously, the hatching time was also equally influenced by photoperiod and temperature. It could be observed from the present study that the minimum and maximum hatching times reported were 18.2 h and 29.6 h respectively (Table 3). At the completely dark photoperiod (0 h), hatching time registered the lowest value compared to the medium (12 h) and maximum level (24 h) (Fig. 2a,b,c). This indicates that light had a triggering effect on hatching of the cysts^20^. The REML ANOVA table revealed that the quadratic effect of photoperiod and temperature played a major role in the variation observed in hatching time (Table 1). The individual effects of photoperiod, temperature and salinity on hatching time were previously reported by unifactorial experiments^7^. In the Great Salt Lake strain of *Artemia* the hatching time was directly proportional to the temperature of the hatching medium within a range of 25 to 30 °C^21^. However, the multifactorial design in the present study enabled us to elucidate that smaller changes in the value of photoperiod and temperature resulted in relatively greater degrees of change in the magnitude of hatching time which could be attributed to their quadratic effects on hatching time. Further the interaction between the photoperiod and temperature significantly affected the hatching time of *Artemia*. The temperature above 32 °C is considered as a non-lethal heat shock inducing condition which is highly stressful for the developing *Artemia* embryo^6^,^23^, hence this could explain the observed lower hatching time and hatching percentage of the cysts at higher temperature in the present study.

In general, hatching percentage was positively influenced by the combined effects (linear and quadratic) of photoperiod. Even though these effects appear to be negative in case of temperature, the positive effects of temperature-salinity interaction along with salinity effect more than compensates for it. This can be seen by plugging in equation 1, coded values 0.95, 0.46 and −1 obtained corresponding to the optimal values (23.35 h photoperiod, 29.32 temperature and 28 ppt salinity). It is noted here that constant term 85.77 in equation 1 has been obtained not in the absence of three independent variables but at the medium levels of them. An optimal value of 96.8 hatching percentage has been obtained over and above this value due to the contribution of these variables and interactions. Similarly plugging the same coded values for optimum values of independent variables in equation 2, we can observe that the constant term 21.72 (which has been obtained at the medium levels of these variables) has been reduced to an optimal hatching time of 18.94 h. Thus the effects of the significant variables and interactions can be taken as positive when reduction in hatching time is to be achieved.

The REML result of the verification experiment was similar to the REML results of the original experiment, where photoperiod and temperature significantly affected the hatching percentage. The hatching percentage was also influenced by the temperature-salinity interaction. Similarly hatching time was significantly affected by photoperiod and temperature as well as their interaction. The results of single factorial hatching experiment were similar to the main effects of the BBD-SSPD REML analysis.

The overall effect of photoperiod, temperature and salinity on both hatching percentage and hatching time could be elucidated from the desirability ramp (Fig. 3c). In routine hatchery operations, hatching percentage is more important than hatching time because variation in the hatching time within a desirable range (18–23 h) is acceptable. However, any reduction in the hatching percentage would reduce the number of nauplii available for feeding finfish or shellfish larvae, and consequently increase the quantity of cysts required for hatching. This in turn would affect the economic viability of hatchery operations. Therefore in the present study, the importance scores of 5 and 4 (as indicated by + sign in Table 2) were given to the hatching percentage and hatching time, respectively.

During the calculation of desirability function, the goal settings of hatching percentage and hatching time were set as maximum and minimum, respectively to accomplish maximal hatching output within minimum time (Table 2). This goal setting had ensured the maximum hatching percentage with minimum operating cost. As the hatching time increases, the cost of power required to maintain continuous aeration, illumination and temperature would also increase leading to greater operational cost. As the desirability function increases from 0 to 1 the yield of the responses is either maximized or minimized as in the case of hatching percentage or hatching time, respectively. The maximum desirability of 0.928 was attained at 23 h photoperiod, 29 °C temperature and 28 ppt salinity, which resulted in 96% of hatching within 18 h for the *Artemia* cysts (Great Salt Lake origin) used in the present study. These results may however, vary according to the brine shrimp strain used. Therefore our study provides sufficient information on how the hatching optimum changes with unit change in the abiotic factors. It also provides an insight into the relative importance of each factor on hatching and how they interact with each other.

In a conventional full factorial design, responses from all possible treatment combinations have to be recorded for optimization (for instance in a four-factor full factorial experiment, each treatment with three levels, it is possible to have 81 treatment combinations that have to be replicated again for a few times to obtain reliable results (e.g. three replicates result in 243 treatment runs). Practically, such a design is often difficult and expensive to organize, but this could be resolved by selecting a Box Behnken Design (BBD). The BBD was selected in the present study due to the physiological requirement of *Artemia* cysts especially with reference to their hatching process. In the central composite design (CCD, a design commonly used for optimization similar to BBD), if it had been used in the present study, observations had to be recorded for a factorial level of −8 h and 32 h of photoperiod and 19 and 36 °C of temperature. The photoperiod of −8 h and 32 h does not have any practical significance. At 36 °C and above the *Artemia* cysts stop metabolic activity and revert to a state of quiescence^18^. Therefore, the CCD model was not chosen even though it had been considered as the best design to optimize the responses of interest in this study. The best alternative available for studying the three factors each with three levels was BBD, which is a very popular industrial design. Its acceptability could be attributed to the simplicity and the least cost of the design with minimal treatment combinations^12^ (18 in this study compared to 81 in conventional design). In addition to the functional relationship, the response surfaces could be represented graphically, which leads to a better understanding of the interaction between the abiotic factors and the responses. So this method can be adopted in similar studies on the hatching process of various animal eggs.

Traditionally the BBD experiments were run in a completely randomized manner to obtain reliable estimates of the treatment effects without any bias. However, in the present study, the treatment combinations selected using BBD were arranged in a split-split plot manner to study the hatching process of eggs. By applying randomization restriction in the split-split plot design used in the present study the equipments required was reduced to two tube lights, nine heaters and nine aquarium tanks, which considerably reduced the cost of experimentation. It also minimized the space required for conducting the experiment. So the experimental outputs could be accomplished with minimum cost and apparently better efficiency as previously reported^24^. The present study is the first report to apply a combination of BBD and split-split plot design (SSPD) to study the hatching process of *Artemia* that considerably reduced the cost of the experiment and also produced equally competent results.

Optimization of the multiple responses is very essential for successful operation of an aquaculture hatchery. Contour plots and 3D response surface plots are useful in arriving at a conclusion about the optimum conditions of the single response only. For a desirable product development, several responses may have to be optimized simultaneously. Some responses have to be maximised or minimised while others may be kept within a range. This could be accomplished using a desirability approach where optimum levels of each factor were simultaneously determined to achieve multiple goals. By examining contour plots the influence of each factor on single response could be elucidated. The most commonly used approach is the graphical optimization hence individual contours were overlaid. The optimum operating conditions could be determined by visual inspection of the overlaid contour plot (Fig. 3d). When the number of factors is more than three, graphical optimization becomes difficult. In such cases, the application of desirability function could be useful.

It is practically impossible to keep all the environmental conditions at constant level in aquaculture operations. Even though it is possible to control the environment using equipments to a certain extent, this would considerably increase the cost of operations. The overlay plot would then be a handy tool for hatchery operators in such situations. The plot represents the common area of exploration that ensures minimum desirable output. For example if the environmental temperature is decreasing because of weather conditions, the hatchery operator can visually inspect the overlay plot and find out at what point the heater has to be switched on to avoid losses. In real world situations, this would help hatchery operators or technicians and it does not require any expertise in statistics or animal physiology to adjust the operating conditions according to the changing environment. Essentially this means that a management decision can be taken by simply observing the overlay plot.

## Conclusion

Multiple factor interactions often pose many practical and economic constraints in randomly organizing all the treatment combinations across the experimental units. This could be solved by adopting a novel approach combining Box-Behnken Design and a Split-split-plot Design (BBD-SSPD) that could facilitate efficient organization of a multifactorial trial in a cost-effective manner without losing any degree of accuracy. This method was used to optimize the hatching process of brine shrimp *Artemia* cysts in this study, which clearly established that the interactions of photoperiod, temperature and salinity had affected the hatching process of cysts. It was found that the most important factors that controlled the hatching process were photoperiod (23 h) and temperature (29 °C). This approach could also be applied in other designs like full factorial design and central composite design using split-split plot arrangement. The model developed would be useful in optimizing the production process of several economically important aquatic species and in the poultry industry.

## Material and Methods

### Box- Behnken Design

In this study, the Box-Behnken Design (BBD) was adopted to evaluate the combined effect of photoperiod, temperature and salinity on hatching percentage and hatching rate of *Artemia* cysts^25^. In BBD, treatments (levels) of a factor are evenly spaced (for example treatments of photoperiod were 0, 12 and 24 h with an equal difference of 12 h between the treatments). The number of treatment combinations in BBD was selected using equation 3^26^. The Box-Behnken Design matrix used in the present study is shown in Table 3. Three factors namely photoperiod, temperature and salinity each with three equally spaced treatments and a central point (a treatment combination where the levels of all the three factors were medium) resulted in 13 treatment combinations, which were selected using Design Expert (version.9.0.6.2) software. Central points were replicated six times resulting in 18 experimental runs (Table 3).

The number of experimental runs (N) needed for the development of a BBD is:


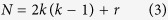


where, k is the factor number and r is the number of replications in central points.

The variables are coded with −1, 0 and +1 for low, medium and high levels of factors. In this study magnitude of the difference between the treatments of each factor was 12, 5 and 5 units for photoperiod, temperature and salinity, respectively. This variation would affect the analysis but was solved using coding which reduced the effect of variables of different orders of magnitude in the analysis^27^. The lower (−1), intermediate (0) and higher (+1) levels of the photoperiod were 0 h, 12 h and 24 h; that of temperature were 22 °C, 27 °C and 32 °C; and that of salinity were 28, 33 and 38 ppt, respectively.

### Split-Split Plot Design

**Figure 4:**
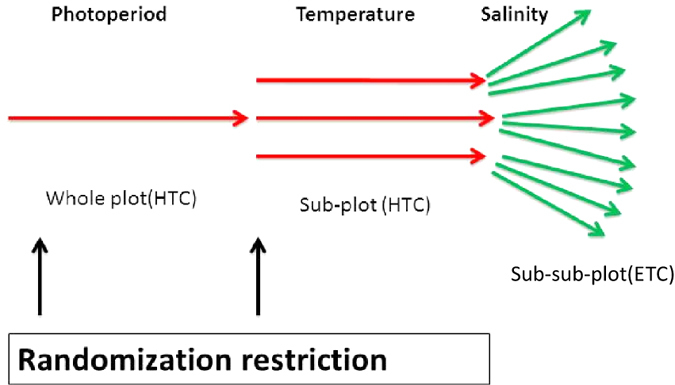
Demonstration of randomization restriction in a single whole plot and arrangement of whole plot, sub plot and sub-sub plot in split-split plot design in a full factorial design. There are two randomization restrictions, photoperiod and temperature (HTC factors) hence the design is Split-Split Plot Design (SSPD). One level of photoperiod (whole plot) is selected and three levels of temperature (sub plot) was randomly allotted within the whole plot. Salinity is the sub-sub-plot factor which is easy to change (ETC). For this, within each of the three levels of temperature sub plot, three levels of salinity were randomly allotted.

**Figure 5:**
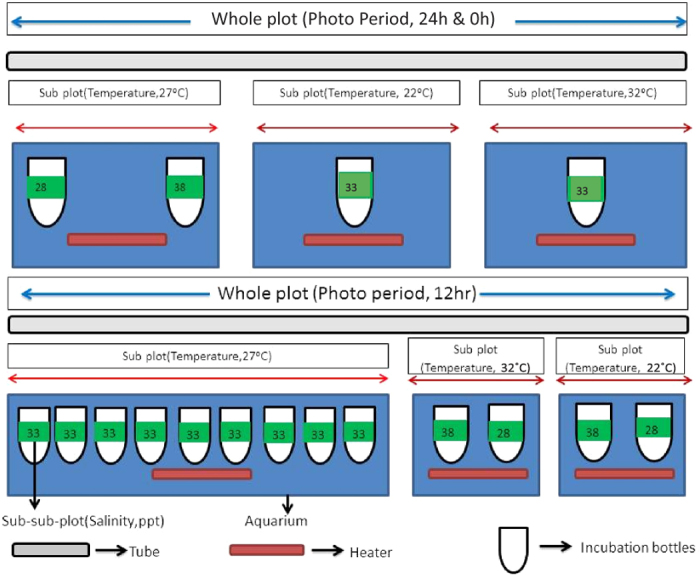
Experimental set up −0 h, 12 h, and 24 h photoperiod whole plots. All aquarium used in the actual experimental set up was having equal dimension, for the illustration purpose aquarium sizes were adjusted. In 12 h whole plot the all the replicates of central point are arranged in the either side of the same aquarium.

The 18 treatment combinations selected using BBD were allocated to the individual experimental units using the SSPD. In general, split plot design is characterized by randomization restriction where factors (photoperiod, salinity or temperature) are not allocated in a random order to the experimental units. The hard-to-change (HTC) factor (the factor which is difficult to change in a split-plot design) is not allocated to individual experimental units in a random order, but the experimental units are grouped into blocks and HTC factors are randomly allotted to the different blocks, known as the whole plot (see Figs 4 and 5). The number of blocks or whole plots is equal to the number of replications of first HTC factor multiplied by the number of treatments of first HTC factor. Each whole plot is subdivided into subplots which consists of a group of experimental units and within each sub plot, the selected levels (treatments) of the second HTC factor is randomly allotted. The number of subplots within a whole plot is equal to the number of treatments of second HTC factor. The randomization restriction can be extended to any number of HTC factors but in this case, it was applied to two factors (i.e. two HTC) and therefore, the design is a split-split-plot design (SSPD). The treatments of the easy-to-change (ETC) factor are then randomly allotted to the sub-sub plots. The number of sub-sub plots is equal to the number of treatments of ETC factor selected using BBD^2^,^11^.

In the present study, photoperiod and temperature were set as HTCs and salinity was the ETC. The whole plot, sub-plot and sub-sub plot were in the order of photoperiod, temperature and salinity (Figs 4 and 5) with three treatments each for photoperiod (0 h, 12 h and 24 h with a constant illumination of 2000 lux), temperature (22 °C, 27 °C and 32 °C), and salinity (28 ppt, 33 ppt and 38 ppt). The quadratic equation for a SSPD can be written as


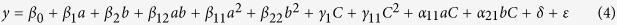


where a, b and C represent photoperiod, temperature and salinity, respectively; and 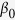 is the constant coefficient (intercept). The β terms include HTC variables (represented in small letters), the γ terms involve ETC variables (represented in capital letters) and the α terms involve both HTC and ETC variables. The β_1_, β_2_ and γ_1_ are slope or linear effect of input factor a, b and C respectively. The β_12,_ α_11_ and α_21_ are the linear interaction effects of input factors. The β_11_, β_22_ and γ_11_ represent quadratic effect of the input factors. The δ is the whole plot error and ε represents the subplot errors^11^. Design and analysis of the RSM model was carried out using Design expert (version 9.0.6.2) software.

### Experimental setup

Firstly, each whole plot (consisting of a set of aquarium tanks (three aquariums) each of 50 L capacity) was allotted with one level of the first treatment (i.e., photoperiod) thus giving three whole plots in total. Within a wholeplot each aquarium tank was randomly allotted with a level of the second treatment i.e., temperature. Thus the three aquariums (each with water of three different temperatures) formed the three sub plots of a single whole plot (or same photoperiod). The bottles each of 1000 ml capacity and with conical bottom were placed in each aquarium and they were randomly allocated with the selected levels (using BBD, Table 3) of the third treatment i.e., salinity (Fig. 5).

The experiment was conducted in an air-conditioned room where the room temperature was maintained at 20 °C. Photoperiods of 12 h: 12 h and 24 h: 0 h (light: dark) were maintained using two fluorescent tube lights (2000 lux). The darkness for 0 h and 12 h photoperiods was created by covering the tanks with black plastic sheets. The temperature of the water was maintained at 22 °C, 27 °C and 32 °C using aquarium heaters with thermostat (Maibo, China with maximum variation of ±1 °C. The different salinities were prepared using synthetic sea salt (Instant Ocean, USA). The plastic bottles were filled with 600 mL of water with the required salinity and they were kept immersed in the glass aquarium (50 L) filled with freshwater and clipped to the side walls.

### Hatching of *Artemia*

*Artemia* cysts (Great Salt Lake strain; INVE, Thailand) stored at 4 °C were hatched according to the standard procedure outlined earlier^17^. In short, 1 g cysts initially hydrated in freshwater for one hour were decapsulated using 4% sodium hypochlorite solution. The decapsulated cysts were incubated in the plastic bottles (600 mL) at different salinities with continuous aeration.

Three single factorial experiments were conducted to check the individual effect of photoperiod, temperature and salinity. In the first experiment treatments were 0, 12 and 24 h photoperiod at 27 °C and 28 ppt salinity. In the second experiment treatments were 22, 27 and 32 °C temperature at 24 h photoperiod and 28 ppt salinity. In the last experiment the treatments were 28, 33, 38 ppt salinity at 24 h photoperiod and 27 °C temperature. Hatching procedure described earlier was also followed in the unifactorial experiments too.

### Determination of hatching percentage and hatching time

Six subsamples, 250 μl each were collected from a single bottle after 12 h incubation and the number of nauplii (N), umbrella (U) and embryo (E) stages were counted. Sampling was continued every 2 h till the hatching percentage became constant. Hatching percentage (H%) was calculated as follows:


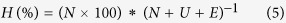


where N is the number of nauplii stages, U is the number of umbrella stages and E is the number of embryo stages in the sample.

The time related variable, hatching time was calculated as the time required for attaining 90% hatching and was estimated using probit analysis in SPSS ver.16.0 software from the hatching percentage data enumerated at different time points.

### REML analysis

The normality was checked using normal plot of residuals to ensure that the distribution was normal. In BBD-SSPD, the observations obtained from the whole plot and sub plot are not independent due to the presence of compound symmetric error structure. Since the goal of the BBD-SSPD is to elucidate confounded factors and their interaction to produce an effect, the use of generalized linear models estimation methods will give results that fall short of expectation. Therefore, the Restricted Maximum Likelihood (REML) approach was adopted to obtain unbiased estimates of variance parameters. Generalized Least Squares (GLS) were used to calculate factor effects and Kenward-Roger’s method was then used to produce F-tests and the corresponding P-values^28^,^29^. After performing the REML analysis, the non-significant terms could be eliminated to improve the predictability of the model by using a stepwise regression procedure. We adopted a backward stepwise regression (with p values at α = 0.1) as it was useful in circumstances when the number of variables in the model was optimum and few variables had to be eliminated for fine tuning of the model to ensure better prediction. This process started with all variables in the model and removed one variable at a time based on the F- statistic.

### Multiple response optimization using desirability function

The optimal output of any process can be achieved through simultaneous optimization of multiple responses. In this study, multiple responses were optimized using the desirability function as proposed by Harrington in 1965^30^ and modified by Derringer and Suich in 1980^31^. It is a statistical procedure that can be used in any second order response surface designs like BBD, in which multiple goals can be set simultaneously and the desirable product quality can be ensured. Desirability is an objective function which converts each individual response into a single desirability function ranging from 0 to 1. A value of zero for desirability function indicates the least desired hatching output and a value of one represents the most desired hatching output. The most desired hatching output in this study was measured in terms of maximum hatching percentage and minimum hatching time. As a first step, individual desirability function for hatching percentage and hatching time was calculated and a simultaneous objective function, a function which combines the individual desirability function was calculated by finding geometric mean of all individual desirability functions. The formula used for desirability (D) is as follows:


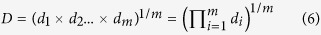


where 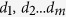 represent individual desirability function and m the number of responses. To achieve the goal of maximising hatching percentage and minimizing hatching time the desirability function was modified by differential weighting in the function to provide added emphasis to target variables i.e., maximum or minimum according to the subject matter knowledge. In present study nil value is given for the input variables and value one is given for responses^11^. Weights range from a minimum of zero to a maximum of ten. With a weight of one, desirability function varies linearly. Any value below one gives less importance to that particular goal during calculation of the multiple desirability function while weights above one adds more importance to that goal. The desirability function was calculated based on the settings given in Table 2. For the graphical optimization, the minimum hatching percentage and maximum hatching time selected were 90% and 23 h, respectively. This setting ensured economical operation of the hatching process.

## Additional Information

**How to cite this article**: Arun, V. V. *et al*. Multi-response optimization of *Artemia* hatching process using split-split-plot design based response surface methodology. *Sci. Rep.* **7**, 40394; doi: 10.1038/srep40394 (2017).

**Publisher's note:** Springer Nature remains neutral with regard to jurisdictional claims in published maps and institutional affiliations.

## Figures and Tables

**Table 1:** REML Analysis for response surface quadratic model of hatching percentage.

Fixed Effects				
Source	Term	Error	F	p-value
	df	df	Value	Prob > F
**REML Analysis for response surface using a quadratic model of hatching percentage.**				
Whole-plot	4	7.58	193.08***	<0.0001
a-Photoperiod	1	7.35	612.74***	<0.0001
b-Temperature	1	8.04	12.38**	0.0078
a ^ 2	1	7.43	86.46***	<0.0001
b ^ 2	1	7.43	48.95***	0.0002
Subplot	3	8.24	5.41*	0.0240
C-Salinity	1	6.93	4.23	0.0791
bC	1	8.04	5.93*	0.0408
C ^ 2	1	7.43	6.65*	0.0348
**Removed**				
ab			0.037	0.8543
aC			0.090	0.7729
Significant codes: ‘***’ 0.001 ‘**’ 0.01 ‘*’ 0.05				
**REML Analysis for response surface using a quadratic model of hatching time**				
Whole-plot	5	5.87	147.51***	<0.0001
a-Photoperiod	1	8.36	245.35***	<0.0001
b-Temperature	1	10.00	485.28***	<0.0001
ab	1	10.00	13.39**	0.0044
a ^ 2	1	4.23	37.24**	0.0030
b ^ 2	1	5.87	27.29**	0.0021
Subplot	2	9.81	1.57	0.2558
C-Salinity	1	9.56	1.040E-004	0.9921
bC	1	10.00	3.15	0.1065
**Removed**				
C ^ 2			0.01	0.9209
aC			0.22	0.6504
Significant codes: ‘***’ 0.001 ‘**’ 0.01 ‘*’ 0.05				

**Table 2:** Desirability analysis of multiple responses: Goal settings and corresponding importance and weight of each variable and responses.

Factor	Goal	Goal settings	Importance	Weight
Photoperiod (h.)	In range	0–24	nil	nil
Temperature (°C)	In range	22–32	nil	nil
Salinity (ppt)	In range	28–38	nil	nil
Response				
Hatching percentage (%)	Maximum	Maximum	+++++(5)	1(upper)
Hatching time (h.)	Minimum	Minimum	++++(4)	1(lower)

**Table 3:** Box-Behnken Design matrix and experimental responses.

Run	Factor 1	Factor 2	Factor 3	Response 1	Response 2	wholeplot
	a:Photoperiod	b:Temperature	C:Salinity	Hatching percentage	Hatching time	
	h	degree Celsius	ppt	%	h	
1	1	1	0	84.9	18.2	I (24 h)
2	1	0	−1	96.3	20.5	
3	1	0	1	96.8	21.2	
4	1	−1	0	91.2	26.3	
5	0	1	1	75.2	19.4	II (12 h)
6	0	−1	1	82.5	26.2	
7	0	0	0	86.1	22.1	
8	0	1	−1	85.0	19.1	
9	0	0	0	85.3	21.9	
10	0	0	0	84.1	21.9	
11	0	−1	−1	83.1	27.5	
12	0	0	0	88.2	22.1	
13	0	0	0	87.4	21.7	
14	0	0	0	86.0	21.0	
15	−1	1	0	48.1	24.8	III (0 h)
16	−1	0	−1	63.2	25.8	
17	−1	0	1	61.1	26.2	
18	−1	−1	0	55.2	29.6	
